# Subtropical Mode Water south of Japan impacts typhoon intensity

**DOI:** 10.1126/sciadv.adi2793

**Published:** 2023-09-13

**Authors:** Eitarou Oka, Shusaku Sugimoto, Fumiaki Kobashi, Hatsumi Nishikawa, Sachie Kanada, Tomoe Nasuno, Ryuichi Kawamura, Masami Nonaka

**Affiliations:** ^1^ Atmosphere and Ocean Research Institute, The University of Tokyo, Kashiwa, Japan.; ^2^ Department of Geophysics, Graduate School of Science, Tohoku University, Aoba-ku, Sendai, Japan.; ^3^ Department of Maritime Systems Engineering, Tokyo University of Marine Science and Technology, Tokyo, Japan.; ^4^ Institute for Space-Earth Environmental Research, Nagoya University, Furo-cho, Nagoya, Japan.; ^5^ Japan Agency for Marine-Earth Science and Technology, Yokohama, Japan.; ^6^ Typhoon Science and Technology Research Center, Yokohama National University, Yokohama, Japan.; ^7^ Department of Earth and Planetary Sciences, Faculty of Science, Kyushu University, Fukuoka, Japan.

## Abstract

Subtropical Mode Water (STMW), characterized by vertically uniform temperature of ~17°C, is distributed horizontally over 5000 kilometers at the 100- to 500-meter depths in the subtropical North Pacific Ocean. Its formation and spreading fluctuate in relation to the Pacific Decadal Oscillation and the Kuroshio path variation, but the feedback from STMW on the sea surface temperature (SST) and the overlying atmosphere remains unclear. Using Argo profiling float data, we show that STMW south of Japan, whose thickness varies decadally, modulates the overlying thermal structure throughout the year by increasing isotherm uplift with increasing thickness. The STMW-induced decadal temperature change has a magnitude of up to ~1°C and is large in the warm season in the presence of the seasonal thermocline. Furthermore, 50-year observations, together with numerical simulation, show that SST, upper ocean heat content, and typhoon intensification rate have been significantly lower in years with thicker STMW and higher in years with thinner STMW.

## Teaser

A water mass in the ocean interior modulates the near-surface ocean temperature and affects tropical cyclone intensity.

## INTRODUCTION

**Fig. 1.**
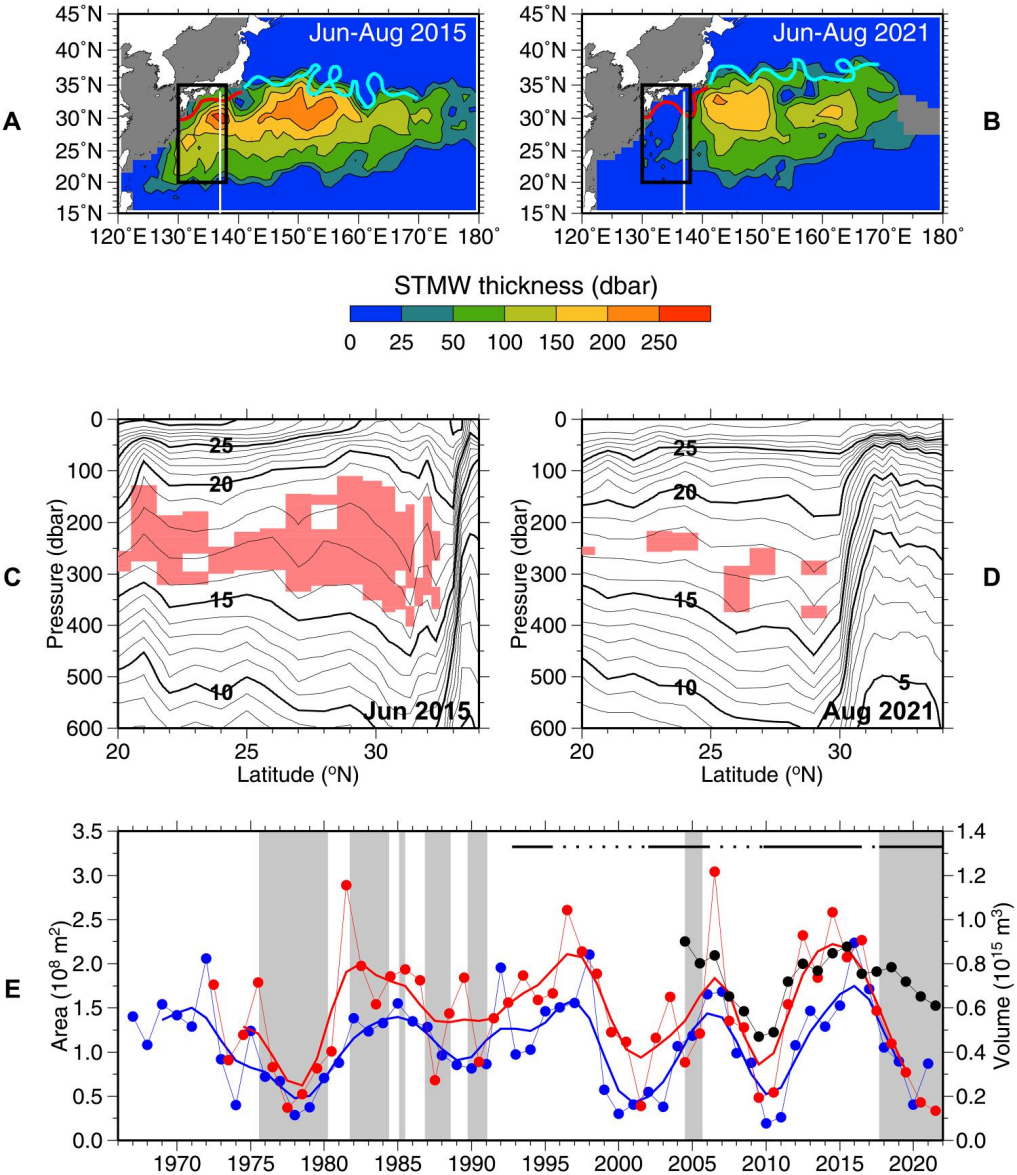
Decadal variability in Subtropical Mode Water (STMW). (**A** and **B**) STMW thickness (*6*) averaged for June–August 2015 (A) and June–August 2021 (B), based on Argo profiles. The black line denotes the study region (20°N to 35°N, 130°E to 138°E). Red and cyan curves represent the positions of the Kuroshio and the Kuroshio Extension (KE), respectively, in July 2015 and July 2021, which are based on satellite altimeter sea surface height data. Note that the Kuroshio took a non–large-meander path in 2015 and a large-meander path in 2021. The white line indicates the 137°E repeat hydrographic section. (**C** and **D**) Distributions of potential temperature (°C) in the 137°E section in June 2015 (C) and August 2021 (D). Pink shading denotes STMW. (**E**) Time series of the cross-sectional area of the STMW in the 137°E section in winter (blue) and summer (red) (*15*). The thin curve with dots denotes the yearly values, and the thick curve represents values smoothed with a 1-3-4-3-1 filter. The black curve with dots represents the total STMW volume based on Argo profiling float data (*6*). Solid (dotted) bars indicate stable (unstable) periods of the KE based on satellite altimeter sea surface height data (*13*, *14*). Gray shading denotes large-meander periods of the Kuroshio.

In the western subtropical gyre of the North Pacific Ocean, Subtropical Mode Water (STMW) (*1*) appears within the upper permanent thermocline as a distinct vertically uniform layer with a potential temperature of 16.0° to 19.5°C (*2*), characterized by its low potential vorticity. It is formed as deep-winter mixed layers south of the Kuroshio and the Kuroshio Extension (KE; Fig. 1, A and B) (*3*). The colder variety of STMW (<18°C) that accounts for more than 80% of the total volume of STMW is formed south of the KE, i.e., east of 140°E, while the warmer variety (> 18°C) is formed west of 150°E, mainly in the region south of Japan west of 140°E (*4*). After spring, both varieties are subducted and advected southwestward as far south as 20°N and as far west as the western boundary, transporting temperature/salinity anomalies and substances such as carbon dioxide and nutrients (*2*, *5*–*7*).

The formation rate of the colder variety of STMW south of the KE fluctuates decadally (*6*, *8*, *9*) in association with the Pacific Decadal Oscillation (PDO) (*10*) and the resultant decadal variability of the KE between stable and unstable states (*11*–*14*). This dominates the horizontal extent, thickness, and volume of STMW over the western subtropical gyre (*6*, *9*, *15*). From 1992 to 2016, the STMW formation increased during stable KE periods of 1992–1994, 2002–2005, and 2010–2016 and decreased during unstable KE periods of 1995–2001 and 2006–2009 (Fig. 1E).

The spreading of STMW is also modulated by the Kuroshio path variations south of Japan (Fig. 1, A and B) (*5*, *16*, *17*). In August 2017, the Kuroshio large meander reappeared after a long absence since 1991 (except for a 1-year period in 2004–2005) and has persisted until now, which is the longest period observed (Fig. 1E) (*18*, *19*). Since then, the westward advection of the colder variety of STMW from the region south of the KE to the region south of Japan has been hindered, possibly because STMW cannot pass over a bathymetric gap of the meridional Izu-Ogasawara Ridge near 30°N, 140°E (*7*) and is dissipated because of enhanced diapycnal mixing over the ridge south of 30°N (*4*, *20*–*22*). As a result, the thickness of STMW south of Japan decreased from its peak around 2015 to its lowest level in 2021 (Fig. 1), although the total volume of STMW did not decrease notably during this period (black curve with dots in Fig. 1E).

Given the pronounced decadal variability in the STMW formation and spreading, the next important question is the feedback from STMW on the overlying layers and the regional climate. Previous studies have considered upward transfer of temperature anomalies or substances from the STMW layer either by the reemergence (or remote reemergence) mechanism (*23*–*26*), in which subducted mode waters are reentrained into the mixed layer in the following winters, or by vertical diffusion (*27*, *28*). On the other hand, a recent analysis (*29*) of summer observations at the 137°E repeat hydrographic section south of Japan (Fig. 1, A and B) (*30*) showed that thicker STMW is associated with a shallower seasonal thermocline on decadal timescales, as exemplified in Fig. 1 (C and D), likely because STMW uplifts the overlying isotherms through the baroclinic adjustment mechanism (*31*). Such decadal variability in the seasonal thermocline depth in summer is expected to modulate the upper ocean heat content above STMW and may affect the development and decay of typhoons moving northward in the region south of Japan, because high upper ocean heat content, or sea surface temperature (SST), is favorable for tropical cyclone intensification (*32*–*36*).

Because STMW exists all year round, it is of interest to examine how the uplifting effect of STMW affects the overlying stratification, not only in summer but also in the other seasons, and impacts the typhoon intensity. In this study, we analyze Argo profiling float data to demonstrate that STMW south of Japan modulates the overlying thermal structure throughout the year, particularly in the warm season when the seasonal thermocline is present. We also examine long-term SST, the tropical cyclone best track, and the 137°E repeat hydrographic section data to show that SST, upper ocean heat content, and tropical cyclone intensification rate (TCIR) south of Japan in the warm season were significantly lower in years with thick STMW and higher in years with thin STMW. We further perform numerical simulation for Typhoons Lan (2017), Hagibis (2019), and Chan-hom (2020) to demonstrate that their central pressure would increase by up to 3.1 to 8.7 hPa if STMW south of Japan had a thickness equivalent to what it was in 2015 when it was at its peak.

## RESULTS

### Impact of STMW on the overlying thermal structure

We first analyzed Argo profiling float data (*37*) in the region south of Japan and west of the Izu-Ogasawara Ridge (20°N to 35°N, 130°E to 138°E; Fig. 1, A and B) from 2010 to 2021, which corresponds to 1 cycle of decadal STMW variability (Fig. 1E). Using the Argo data, the correlation between the STMW thickness and potential temperature at each depth between 0 and 600 dbar was calculated for each month (anomalies from the 12-year monthly mean at each location were used for both the STMW thickness and potential temperature; see Materials and Methods for details). Here, a positive (negative) correlation means that the potential temperature at that depth increases (decreases) as STMW in the water column thickens.

**Fig. 2.**
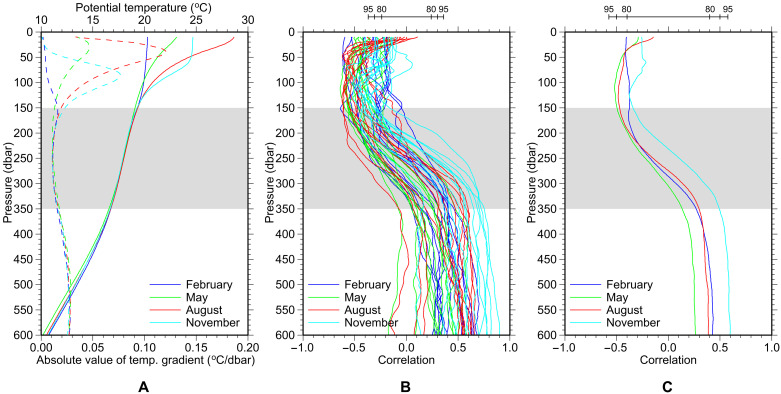
Relationship between the Subtropical Mode Water (STMW) thickness and the thermal structure. (**A**) Average vertical profiles of potential temperature in the study region in February, May, August, and November, based on Argo profiles containing STMW thicker than 100 dbar in 2010–2021 (solid curve) and their vertical gradient (dashed curve). STMW is characterized by a small gradient centered at 150 to 350 dbar (gray shading). (**B**) Correlation between the STMW thickness anomalies and potential temperature anomalies at each depth in the study region in February, May, August, and November of 2010–2021 (total of 48 months), based on Argo profiles. The bar above the panel denotes the 80, 90, and 95% confidence intervals. (**C**) As for (B) but for February, May, August, and November throughout 2010–2021.

Considering the STMW layer centered at 150 to 350 dbar (Fig. 2A), the correlation changes sign for each month during 2010–2021, being negative above STMW and positive below STMW regardless of the season (Fig. 2B). The deep positive correlations express the well-known tendency that thicker mode waters exist where the permanent thermocline is deeper, e.g., in anticyclonic eddies (*38*, *39*). The shallow negative correlations indicate that isotherms above STMW, including those in the seasonal thermocline in the warm season (Fig. 2A), tend to be shallower where STMW is thicker, implying that STMW modulates the overlying stratification on regional scales through the uplifting effect. Note that the combination of a shallower seasonal thermocline above the thicker STMW and a deeper permanent thermocline below it somewhat resembles mode water eddies (*40*). Our analysis shows that such a relation between STMW and the seasonal thermocline depth on a mesoscale applies to larger spatial scales.

The correlation between the STMW thickness and potential temperature for each month throughout the 12-year period of 2010–2021 is negative and moderate (around −0.5 to −0.25) above STMW and positive and moderate (around 0.25 to 0.6) below STMW in all seasons, indicating that the uplifting effect of STMW also works on decadal timescales (Fig. 2C). The exception is the drop in the correlation at depths less than 30 dbar (i.e., above the seasonal thermocline; Fig. 2A) in August (Fig. 2C), which is also seen in individual years (Fig. 2B). This might be partly due to the relatively coarse vertical resolution of a certain percentage of the Argo profiles, which can lead to inadequate interpolation or extrapolation near the top of sampling depths.

**Fig. 3.**
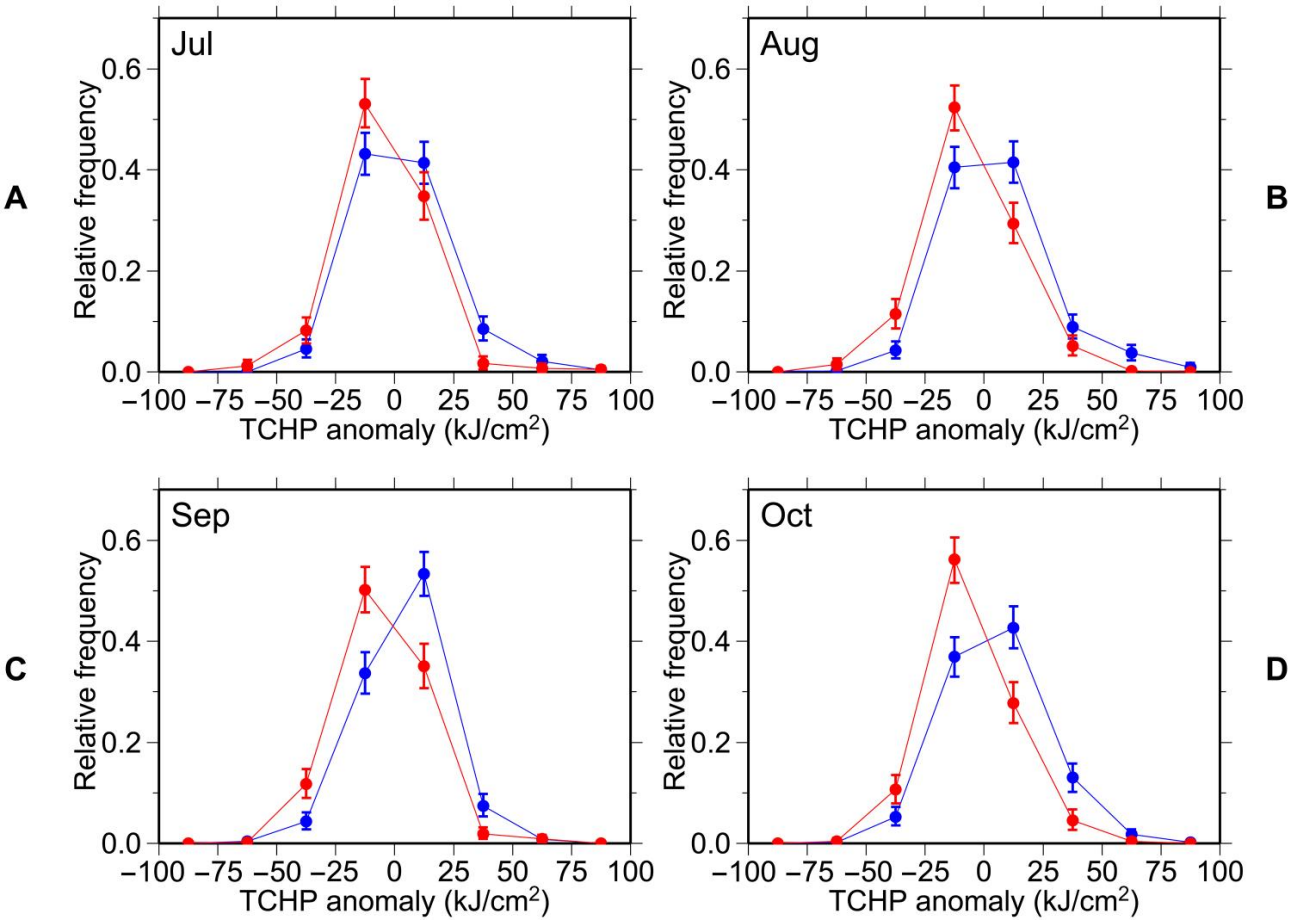
Relationship between the Subtropical Mode Water (STMW) thickness and the upper-ocean heat content. Relative frequency of the tropical cyclone heat potential (TCHP) anomalies in the study region, based on Argo profiles with positive (red line) and negative (blue line) STMW thickness anomalies in July (**A**), August (**B**), September (**C**), and October (**D**) throughout 2010–2021. Here, TCHP anomalies are defined as the anomalies from monthly mean in each 1° grid box (see Materials and Methods). Error bars represent the 95% confidence interval.

The moderate negative correlations within and below the seasonal thermocline in summer mean that the upper ocean heat content above STMW is lower in years with thicker STMW. This may have important implications for tropical cyclone activity, as the summer tropical cyclone heat potential (TCHP), defined as the ocean heat content between the sea surface and the 26°C isotherm (*41*), has been suggested to be more closely related to tropical cyclone intensification than SST (*35*). Our results show that the TCHP computed over the study region exhibited negative (positive) anomalies more frequently where STMW was thicker (thinner) than average (Fig. 3).

Lower upper-ocean heat content associated with thicker STMW in summer is not only likely to suppress tropical cyclone intensification but also likely to cause the development of colder mixed layers in the following winter, as indicated by the moderate negative correlation in February extending up to the sea surface (Fig. 2C). It should be mentioned that winter mixed layers south of Japan are too warm (Fig. 2A) to reach the colder variety of STMW (<18°C), which is advected from the region south of the KE to form the major portion of STMW south of Japan (*4*). In other words, STMW formed south of the KE and advected from there, with its decadally varying thickness, affects the mixed-layer temperature south of Japan in the following winters even without being reentrained into the mixed layer. We propose to call this mechanism the “indirect reemergence” of temperature anomalies.

**Fig. 4.**
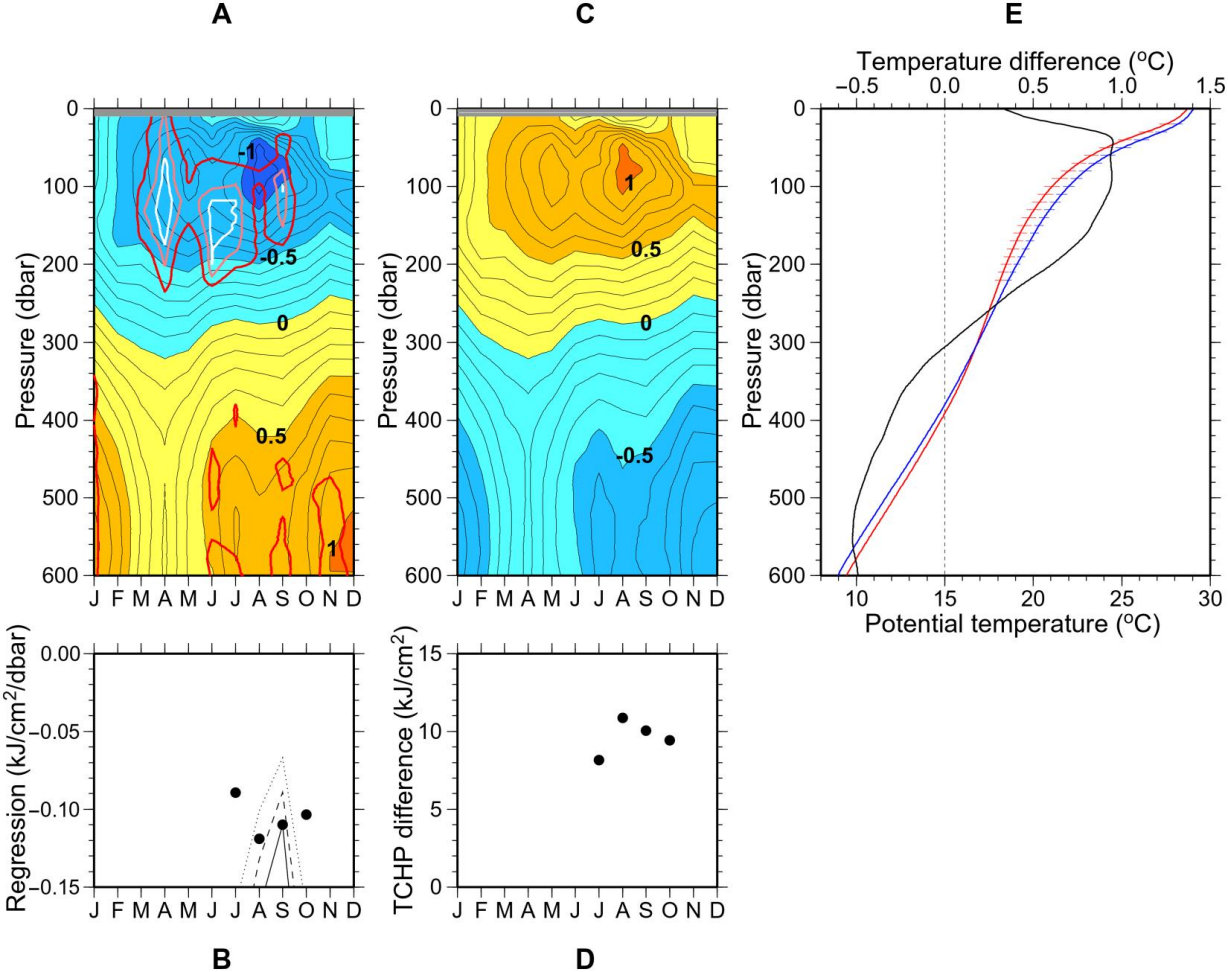
Thermal structure change associated with Subtropical Mode Water (STMW) thickness change. (**A**) Regression coefficients of potential temperature anomalies at each depth on the STMW thickness anomalies (10^−2^°C/dbar) for each month, based on Argo profiles. Red, pink, and white lines indicate that the regression is significant at the 80, 90, and 95% confidence levels. (**B**) Regression coefficients of tropical cyclone heat potential (TCHP) anomalies for each month between July and October, based on Argo profiles. Dotted, dashed, and solid lines denote that the regression is significant at the 80, 90, and 95% confidence levels. (**C** and **D**) Difference in temperature at each depth (°C) for each month (C) and TCHP in July–October (D) associated with the 2021 minus 2015 annual-mean STMW thickness difference. (**E**) Average vertical profiles of potential temperature between 20°N and 30°N at the 137°E repeat hydrographic section in summer in the years with thick STMW (red curve) and thin STMW (blue curve) and their difference (black curve). Horizontal lines in red and blue denote the 95% confidence interval at depths where the values from the two curves are significantly different.

Given the moderate correlations below and above STMW on decadal timescales (Fig. 2C), it is reasonable to calculate the linear regression of potential temperature at each depth over the STMW thickness for each month using the least-squares method (again, anomalies were used for both the STMW thickness and potential temperature; see Materials and Methods). The regression coefficient is negative throughout the year at depths less than 250 dbar and tends to be high at about 50 to 100 dbar in the warm season, reaching a minimum of −1.2 × 10^−2^°C/dbar at 80 dbar in August (Fig. 4A). Such a high negative regression coefficient in the subsurface during the warm season is thought to be associated with the seasonal thermocline, whose vertical shift induced by STMW greatly changes the isobaric temperature. From June to September, low coefficients are found near the sea surface above the area of high negative coefficients. Nonetheless, the regression of TCHP from July to October on the STMW thickness was −0.09 to −0.12 kJ/cm^2^ per decibar, which was significant at the 95% confidence level in September and at the 86% confidence level in August (Fig. 4B). This suggests a possible relationship between TCHP, and hence tropical cyclone intensity, and the STMW thickness in the study region.

As mentioned above, the STMW thickness south of Japan has decreased greatly since around 2015 because of the occurrence of the Kuroshio large meander (Fig. 1). How much has it changed the overlying thermal structure? The annual mean STMW thickness in the study region was 131 dbar in 2015 and 40 dbar in 2021 (fig. S1). The difference of 91 dbar multiplied by the regression coefficient in Fig. 4 (A and B) gives the difference in monthly temperature distribution and summer TCHP between these years in relation to the changes in the STMW thickness (Fig. 4, C and D). The estimated temperature increase from 2015 to 2021 exceeds 0.5°C at depths less than 150 dbar from March to October, except near the sea surface in June to September, and reaches a maximum of 1.1°C at a depth of 80 dbar in August. The summer TCHP has also increased by 8 to 11 kJ/cm^2^ from 2015 to 2021, which is likely to affect the probability of exceeding the critical TCHP values for the tropical cyclone intensification around the study region (60 to 80 kJ/cm^2^) (*36*).

The 12-year Argo data have shown that STMW affects the overlying thermal structure through the uplifting effect, both on regional (Fig. 2B) and decadal (Figs. 2C and 4, A to D) scales. However, because the decadal timescale of the variability is large compared to the duration of Argo float observations (in other words, the degrees of freedom were low), the statistical significance was not necessarily high on decadal timescales. Therefore, to see whether similar changes in the thermal structure due to the decadal variability in the STMW thickness (Fig. 1E) were observed, we also examined the long time series at the 137°E repeat hydrographic section (*30*) during the summer of 1972–2021.

From the 50-year long time series, 6 years with thick STMW and 10 years with thin STMW were selected on the basis of the STMW thickness averaged between 20°N and 30°N (fig. S2; in the following, we focus on the southern part of the study region at 20°N to 30°N, 130°E to 138°E to exclude the direct influence of the Kuroshio path variations from the multidecadal analysis). The potential temperature averaged at 20°N to 30°N in the thin-STMW years was higher than that in the thick-STMW years at depths less than 300 dbar (Fig. 4E). The temperature difference exceeded 0.5°C at 20 to 220 dbar, which was significant at the 95% confidence level, and reached 0.9°C at 30 to 120 dbar. In addition, the average TCHP at 20°N to 30°N was higher in the thin-STMW years (32.0 kJ/cm^2^) than in the thick-STMW years (22.1 kJ/cm^2^). The difference of 9.9 kJ/cm^2^, comparable to values obtained from the Argo profiles (Fig. 4D), was also significant at the 95% confidence level.

The difference in subsurface temperature and TCHP between the thick- and thin-STMW years for the summer 137°E section was significant, while the difference in SST was relatively small (0.34°C) and insignificant (Fig. 4E). This is partly due to diurnal variations but more importantly to seasonal variations associated with the different dates of observations along 137°E, especially in recent years (fig. S3). While the observations at 25°N, 137°E were made in the second half of July in 26 of 50 years, they were scattered between June 11 and September 3, with a mean and SD of July 21 ± 15.7 days.

**Fig. 5.**
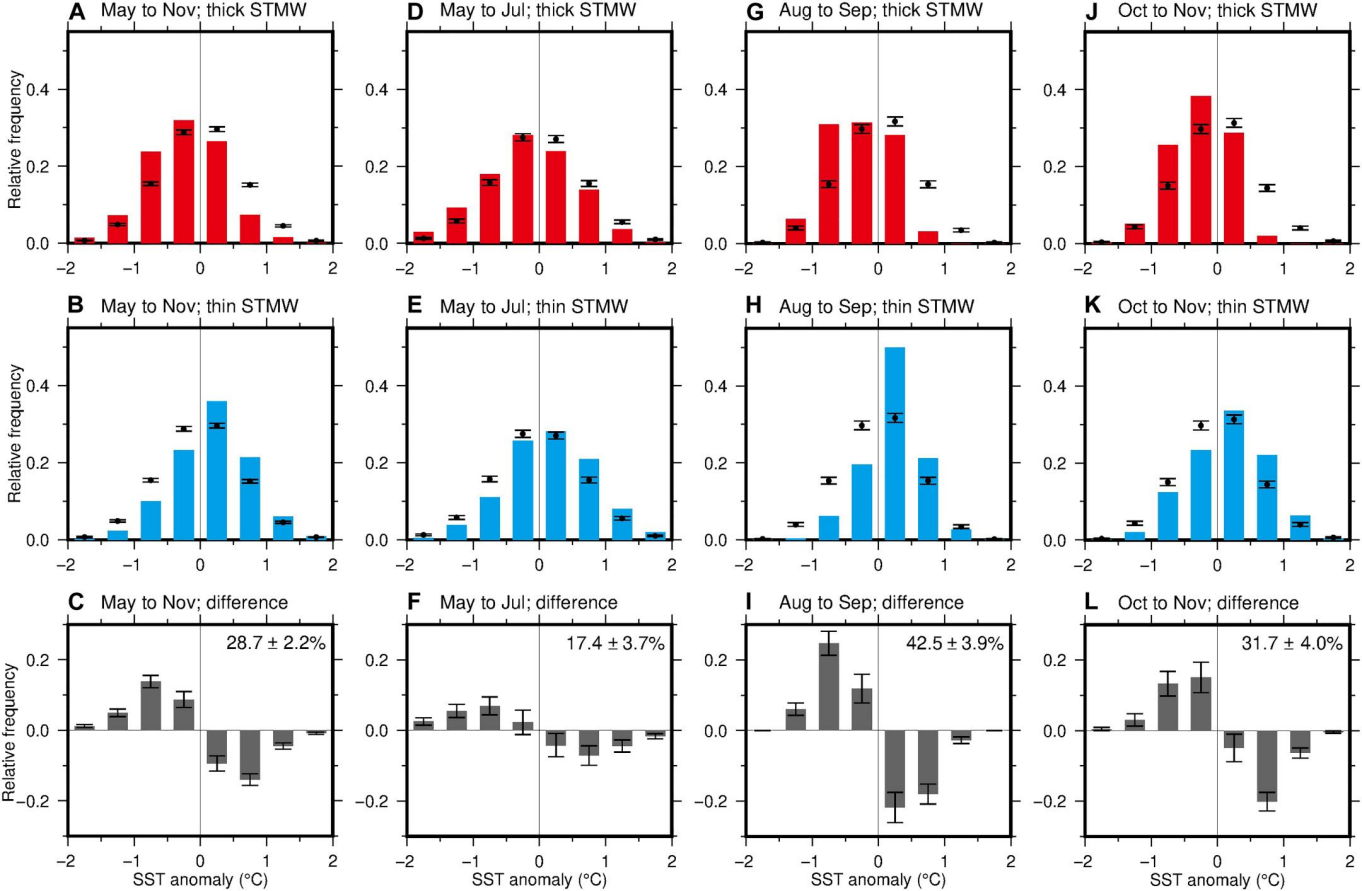
Observed dependence of sea surface temperature (SST) on the Subtropical Mode Water (STMW) thickness. (**A** and **B**) Relative frequency of the SST anomalies over the southern part of the study region (20°N to 30°N, 130°E to 138°E) in the years with thick STMW (A) and thin STMW (B) between 20°N and 30°N at the 137°E section in summer (fig. S2). Black dots with error bars represent the relative frequency and its 95% confidence interval for the entire period from 1982 to 2021. (**C**) Difference in the relative frequency between the years with thick and thin STMW (former minus latter) and its 95% confidence interval. The number at the top right corner denotes the difference in the total relative frequency of the positive or negative SST anomalies between the years with thick and thin STMW and its 95% confidence interval. (**D** to **L**) As for (A to C) but for May–July (D to F), August–September (G to I), and October–November (J to L).

To better distinguish decadal variability from variability associated with these sources, we analyzed National Oceanic and Atmospheric Administration (NOAA) Optimal Interpolation (OI) SST data from 1982 to 2021. For the aforementioned thick- and thin-STMW years calculated at 137°E, SST over the southern part of the study region (20°N to 30°N, 130°E to 138°E) in the warm season was categorized (Fig. 5). During the thick-STMW years, the frequency of the positive (negative) SST anomalies was generally lower (higher) compared to the whole period (Fig. 5A). In contrast, the frequency of the positive (negative) SST anomalies was generally higher (lower) during the thin-STMW years (Fig. 5B), with a clear difference between the thick- and thin-STMW years (Fig. 5C). These trends also hold for individual months, being strongest in August to September, when the tropical cyclone activity in the western North Pacific is at its peak (Fig. 5, D to L). Thus, the long-term observations have demonstrated that both SST and TCHP in the warm season were significantly modulated by the decadal variation of the STMW thickness. This is expected to affect the development and decay of atmospheric convective systems, such as typhoons, through the probability of exceeding the critical SST or TCHP values for tropical cyclone intensification (*33*, *36*, *42*–*46*).

### Impact of STMW on tropical cyclones

**Fig. 6.**
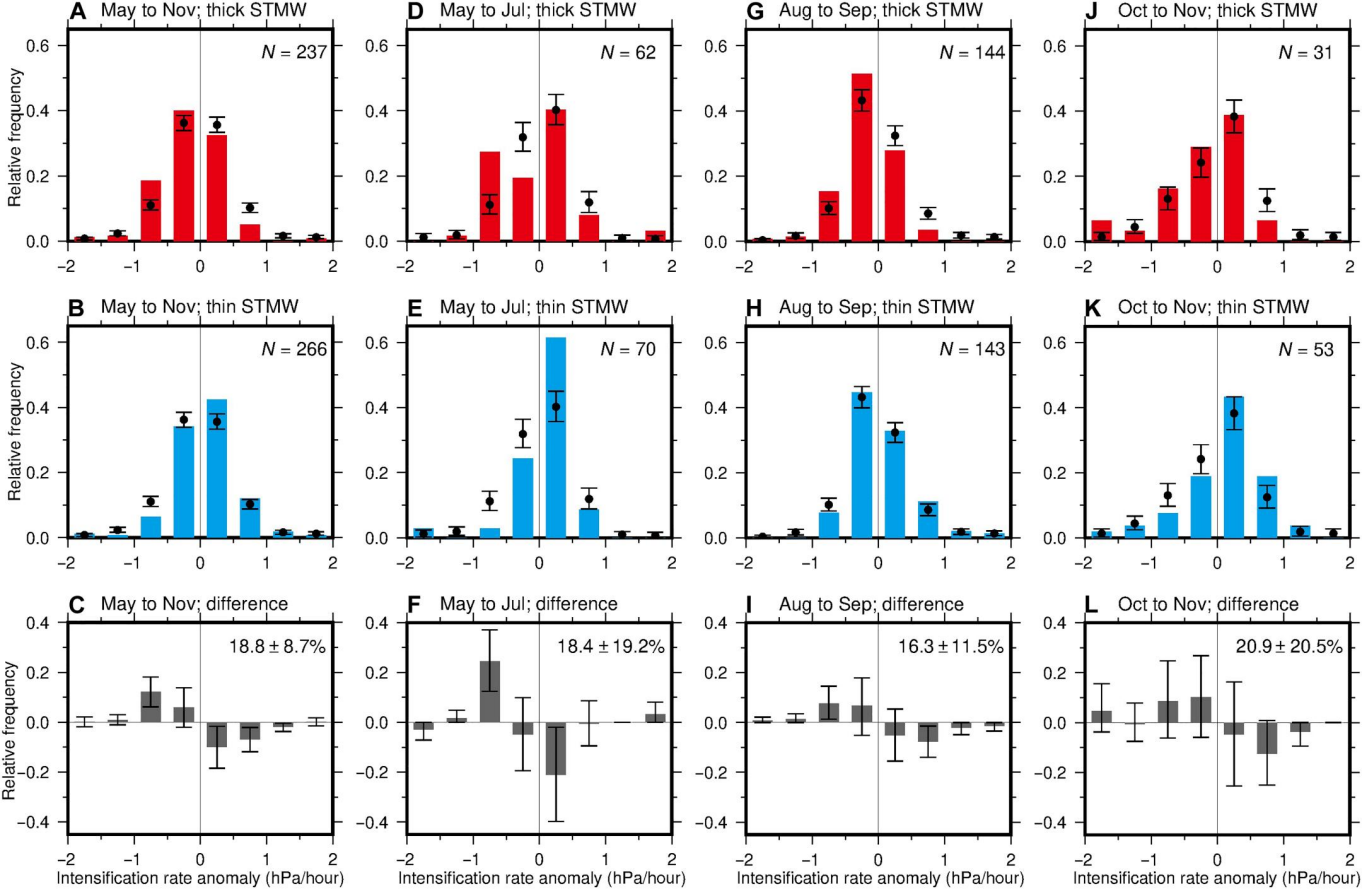
Observed dependence of the tropical cyclone intensification rate (TCIR) on the Subtropical Mode Water (STMW) thickness. (**A** to **L**) As for Fig. 5 but for the TCIR anomalies based on central pressure from the Regional Specialized Meteorological Center (RSMC) Tokyo best track data for the period from 1972 to 2021. The number of data is denoted at the top right corner of each panel in (A), (B), (D), (E), (G), (H), (J), and (K).

To evaluate the influence of STMW thickness on tropical cyclone activity, we analyzed the dataset of the best tracks of tropical cyclones from 1972 to 2021 produced by the Regional Specialized Meteorological Center (RSMC) Tokyo. The TCIR over the southern part of the study region was classified again for the thick- and thin-STMW years at 137°E (Fig. 6). The frequency of the positive (negative) TCIR, which corresponds to a decrease (increase) of pressure with time, tended to be lower (higher) compared to the whole period in the thick-STMW years (Fig. 6A), while it tended to be higher (lower) during the thin-STMW years (Fig. 6B), showing a clear difference between the thick- and thin-STMW years (Fig. 6C). These trends also hold for individual months (Fig. 6, D to L), although the difference between the thick- and thin-STMW years was not significant at the 95% confidence level in May–July and October–November, probably because of small number of data.

**Fig. 7.**
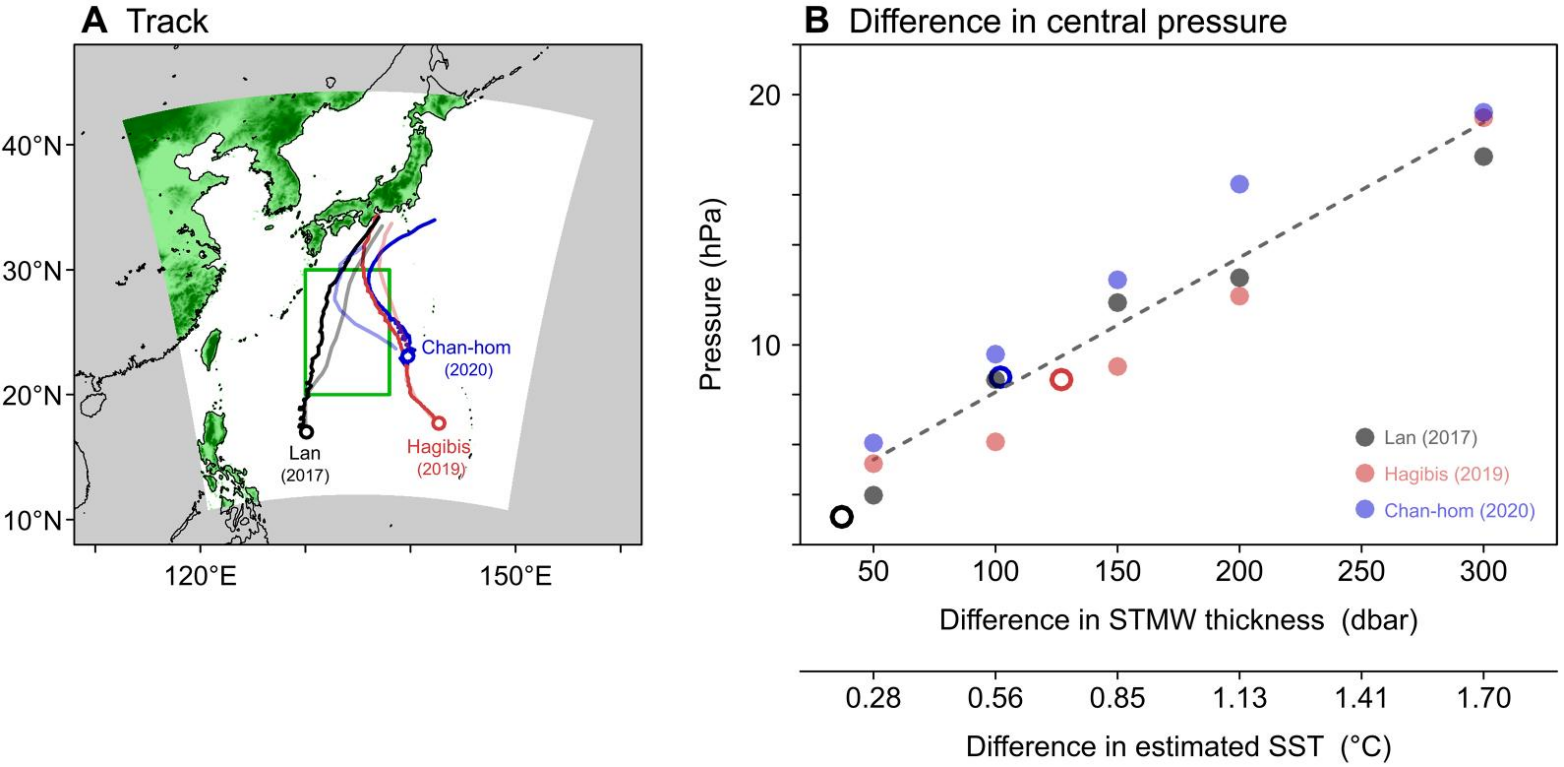
Simulated dependence of typhoon intensity on the Subtropical Mode Water (STMW)–related sea surface temperature (SST) change during the current Kuroshio large-meander period. (**A**) Observed (light color curve) and simulated (dark color curve) tracks of typhoons. Shading denotes the domain of numerical simulation. The green rectangle represents the area with different SSTs. Circles show the locations of typhoons/tropical cyclones at the beginning of simulation. The minimum central pressures of typhoons in the area shown by green rectangle from the observation [control (CTRL) run] were 915 (947) hPa for Lan, 935 (930) hPa for Hagibis, and 965 (958) hPa for Chan-hom. (**B**) Maximum difference in the central pressure of typhoons between each thicker-STMW run and the CTRL run at the same time as a function of the STMW thickness difference (dots), detected for the period after entering the target area and before reaching the southern coast of Japan. The dashed line shows the linear least-squares fit calculated from the dots. Circles denote the result of the same simulation but for the observed STMW thickness difference from 2015. The lower horizontal axis represents the SST difference corresponding to the STMW thickness difference.

To further quantify the impact of STMW-induced SST changes on the tropical cyclone intensity, we performed a suite of numerical simulation of three typhoons using the Japan Meteorological Agency nonhydrostatic model (JMA-NHM) (*47*, *48*) with 5-km horizontal grid spacing. Because the SST response to the STMW thickness varied seasonally (Fig. 4A), we focused on October when the regression at 10 dbar was high (−0.56 × 10^−2^°C/dbar), choosing Typhoons Lan (2017), Hagibis (2019), and Chan-hom (2020) that passed over the study region in October after the current Kuroshio large meander began. Again, STMW south of Japan has shrunk considerably since around 2015 because of the occurrence of large meander (Fig. 1 and figs. S1 and S2), presumably contributing to the increase in SST and near-surface temperature. We therefore simulated the three typhoons under several background oceanic conditions: the control (CTRL) run, in which the observed SST field in October 2017, 2019, or 2020 was prescribed, and the thicker-STMW runs, in which SST anomalies corresponding to STMW thickness anomalies of +50, +100, +150, +200, and +300 dbar were added to the SST field of the CTRL run in the southern part of the study region (20°N to 30°N, 130°E to 138°E; green rectangle in Fig. 7A).

In all runs, the three typhoons were adequately reproduced, passing over the study region northward in similar ways to the observation (Fig. 7A). In addition, the maximum difference in the central pressure between each thicker-STMW run and the CTRL run increased as the STMW thickness anomaly increased, showing a nearly linear relationship (correlation coefficient = 0.95, which is significant at the 95% confidence level; dots in Fig. 7B) We also conducted experiments in which SST anomalies corresponding to the observed STMW thickness differences from October 2015 were used (see Materials and Methods for details), to see how much each typhoon would weaken if STMW had a thickness equivalent to its peak value in 2015. The maximum difference in the central pressure for Lan arising from the STMW thickness difference of 37 dbar was 3.1 hPa, while those for Chan-hom and Hagibis with 102 and 127 dbar reached 8.7 and 8.6 hPa, respectively (circles in Fig. 7B). Considering that pressure reduction associated with typhoons is generally no more than 100 hPa, the systematic changes of 3 to 9 hPa are noticeable. It is thus suggested that the intensity of typhoons is greatly influenced by the STMW-induced decadal SST modulation.

## DISCUSSION

We have shown that the thickness of STMW south of Japan influences the intensity of typhoons by modulating SST and upper-ocean heat content. The STMW-induced changes in typhoon intensity simulated by the numerical atmospheric model in this study were likely overestimated because the model did not account for the SST decrease associated with the passage of the typhoon. Such an SST decrease depends on the speed and size of the typhoon as well as the upper-ocean stratification (*49*–*52*). To quantify these contributions, the next important step will be to perform further numerical experiments of multiple typhoons, not only for the month of October but also for other months. Moreover, instead of using an atmosphere-only model, it will be more appropriate to use a high-resolution coupled atmosphere-ocean model that includes an adequate three-dimensional distribution of STMW.

In terms of ocean dynamics, STMW is known to generate the eastward-flowing subtropical countercurrent (*53*) along its southern boundary by uplifting (not uplifting) isotherms north (south) of the boundary and causing isotherms in the upper 300 m to shallow northward (*54*). The subtropical countercurrent is accompanied by an SST front that affects wind and precipitation, and exerts a notable influence on the atmosphere (*55*). The present study, following Kobashi *et al.* (*29*), has demonstrated a previously unidentified dynamical role of STMW, i.e., through the same uplifting effect, STMW modulates the overlying stratification over its existing area and contributes to the reemergence of temperature anomalies even without being reentrained into the mixed layer.

The present study has also shown that the development of the Kuroshio large meander reduces the STMW thickness on its southwestern side, leading to an increase in SST and upper-ocean heat content and to typhoon intensification in our study region south of Japan. This region is north of the main focus areas of previous statistical analyses on tropical cyclones in the western North Pacific (*43*, *44*). Our findings are consistent with previous case studies around our study region (*45*, *46*) arguing that tropical cyclones’ inner-core processes and air-sea fluxes have an important role in altering TCIRs. The mechanism needs to be further investigated in future studies. Typhoons strengthened by the surface warming caused by STMW in the study region may move northward and make landfall in Japan, bringing extreme wind and rainfall impacts. In addition to the advection of warm water to the coastal regions off the Tokai and Kanto districts, which causes hot and humid summers in the Kanto district including Tokyo (*56*, *57*), the Kuroshio large meander has another influence on social activities during the warm season in Japan.

The uplifting effect of STMW is also expected to affect the near-surface biogeochemistry of the ocean. Oxygen, nutrients, and the acidification rate in the STMW layer at 137°E have fluctuated decadally in association with the PDO-related decadal variability in STMW subduction (*6*, *15*). In particular, apparent oxygen utilization and nutrients increased (decreased) and acidification was sped up (slowed down) during unstable (stable) KE periods when the STMW influx from the formation region south of the KE was decreasing (increasing). This suggests that higher nutrient concentrations in thin STMW are transferred upwards into the euphotic layer enhancing primary production. However, the present study suggests that the thin STMW is accompanied by a deeper nutricline due to a weaker uplifting effect, reducing nutrients and primary production in the euphotic layer. As a result, biogeochemical properties may vary decadally in the opposite phase between the STMW and near-surface layers.

It will be important to examine whether the results of the present study apply to STMW in other areas and basins (*58*). A recent study (*59*) analyzing 30-year ocean reanalysis data showed that the North Pacific STMW subducted and advected southward from the formation region south of the KE also controlled the overlying stratification through the uplifting effect on decadal timescales. STMWs in other basins also show interannual to decadal variations (*60*–*62*). In addition, the North Atlantic STMW has exhibited a notable decline in recent years (*63*), which might have increased upper-ocean heat content and hurricane intensity (*64*).

Last, it is worth mentioning that the recent decline in the STMW thickness south of Japan and the resultant impact on surface ocean-atmosphere processes may serve as a projection for the future ocean (*65*). This is because the winter mixed-layer depth in the STMW formation region has decreased by 6% during the past 60 years and is projected to decline by up to 27 to 40% by 2100 in the models of the Coupled Model Intercomparison Project Phase 6 (*66*). On the other hand, SST south of Japan has increased rapidly in recent years (*67*, *68*), which might be partly because of the STMW reduction. Moreover, SST is projected to increase as a result of anthropogenic greenhouse warming, and so is the maximum intensity of tropical cyclones (*69*–*71*), as the tropical cyclone intensity generally increases as SST increases (*32*, *33*). A future decrease in STMW can amplify the SST increase and the typhoon intensification (*72*–*74*) and lead to more frequent occurrence of marine heatwaves (*75*). In addition, it might amplify future oligotrophication (*76*) by deepening the nutricline. Thus, STMW should be considered one of the key components in future projections for the western boundary current regions and needs to be adequately reproduced in climate models (*77*).

## MATERIALS AND METHODS

### Ocean observation data

We used temperature and salinity data from Argo profiling floats from 2010 to 2021 (*37*). After quality control and discarding temperature and salinity profiles shallower than 900 dbar, we vertically interpolated each profile onto a 1-dbar grid using the Akima spline (*78*). Because most Argo floats do not make measurements at depths less than approximately 5 dbar, which aims to avoid contamination of the conductivity sensor (*37*), we did not analyze Argo data at depths less than 10 dbar. We also used temperature and salinity data obtained along the 137°E repeat hydrographic section (*30*) and optimally interpolated every 20′–1° meridional and 1-dbar vertical interval by the JMA biannually in winter (latter half of January) since 1967 and in summer (around the latter half of July; fig. S3) since 1972, up to 2021. From the temperature and salinity data from Argo floats and the 137°E section, we calculated potential temperature, potential density (σ_θ_), and potential vorticity (*Q*). Here, *Q* is defined as

$$Q=gf\frac{\partial \sigma_{\theta }}{\partial p}$$ (1)

neglecting relative vorticity, where *g* is the gravity acceleration, *f* is the Coriolis parameter, and *p* is pressure.

### Definition of STMW and related properties

STMW in Argo profiles in 2010–2021 was defined as layers with potential temperature of 16.0° to 19.5°C and potential vorticity lower than 2.0 × 10^−10^ m^−1^ s^−1^ (*2*, *4*). The sum of thickness of STMW layers in each Argo profile is considered to be the STMW thickness for that profile. The STMW thickness distribution in Fig. 1 (A and B) was obtained by first averaging STMW thickness in each 1° × 1° grid for each month, then interpolating horizontally (*6*), and lastly averaging for the 3 months between June and August.

STMW in the 137°E section in summer of 2015 and 2021 (Fig. 1, C and D) was defined in the same manner as Argo profiles but that in the long time series during 1967–2021 (Fig. 1E and fig. S2) was defined as areas with potential temperature of 15.0° to 19.5°C and potential vorticity lower than 2.0 × 10^−10^ m^−1^ s^−1^ that were not outcropping to the sea surface (*4*, *15*). The difference in the temperature range between the STMW definitions for 137°E and more contemporary Argo data took the long-term warming of STMW (*79*) into consideration (*4*). On the basis of the STMW thickness averaged between 20°N and 30°N in the 137°E section in summer and a criterion of 1 SD (fig. S2), the 6 years with thick STMW (1981, 1982, 1996, 1997, 2006, and 2015) and the 10 years with thin STMW (1973, 1977, 1978, 1987, 1999, 2001, 2009, 2010, 2020, and 2021) used for Figs. 4E, 5, and 6 were selected.

### Definition of TCHP

TCHP (*41*) is defined as

$$\mathrm{TCHP}=C_{p}\rho_{0}\int_{z_{26}}^{0}(\theta -26)dz$$ (2)

where *z* is the vertical coordinate (positive upward), *z*_26_ is the depth of the 26°C isotherm, ρ_0_ is the reference density (1025 kg/m^3^), *C*_p_ is specific heat of seawater at a constant pressure (4000 J/kg per °C), and θ is potential temperature in °C. TCHP was calculated for Argo profiles, assuming that potential temperature at 1 to 9 dbar was identical to that at 10 dbar, and for the 137°E section data in summer. We did not analyze TCHP in January to June and November to December in relation to the STMW thickness because 37 to 99% of Argo profiles had TCHP values of zero in these months.

### Calculation of correlation and regression using Argo float data

From Argo profiles in the study region (20°N to 35°N, 130°E to 138°E; Fig. 1, A and B) in 2010–2021, we first excluded those with potential temperatures at 400 dbar lower than 12°C to select profiles south of the Kuroshio and the KE where STMW is distributed. We then averaged the STMW thickness, TCHP, and the vertical profile of potential temperature from Argo profiles for each 1° grid box in each month. We subtracted the average STMW thickness, TCHP, and potential temperature from those from each Argo profile in the same grid box and month to obtain anomalies. After discarding anomalies in a grid box in a month with fewer than 10 Argo profiles, the number of anomaly data in the study region in each of 144 months during 2010–2021 was 88.6 ± 27.8.

The correlation coefficients shown in Fig. 2B were calculated for each month during 2010–2021 using the anomalies of STMW thickness and potential temperature as follows. We calculated a correlation coefficient between both anomalies using randomly chosen 30 Argo profiles from that month. After repeating this calculation 10,000 times, correlation coefficients were averaged. The statistical significance of the correlation coefficient was determined by a white noise model using 10,000 samples of 30 random number pairs.

The correlation and regression coefficients shown in Figs. 2C and 4A, respectively, were calculated for each month as follows. We randomly chose one Argo profile for each of 2010–2021 to produce a 12-year time series of the STMW thickness and potential temperature anomalies and calculated a correlation coefficient between them and a regression coefficient of potential temperature anomalies on the STMW thickness anomalies. After repeating this calculation 10,000 times, both coefficients were averaged. The statistical significance of both coefficients was determined by a lag-1 autoregressive red-noise model using 10,000 randomly generated time series. The regression coefficients of TCHP anomalies on the STMW thickness anomalies and their statistical significance (Fig. 4B) were calculated in the same way.

### Histogram of SST and the TCIR

The monthly NOAA OI SST version 2 (V2) data (*80*) were used to examine SST over the southern part of the study region (20°N to 30°N, 130°E to 138°E). They are constructed with a grid interval of 1° based on satellite and in situ observations after December 1981. We used the data from 1982 to 2021 and first calculated the monthly climatology at each grid point. We then computed SST anomalies by subtracting the monthly climatology from each value. The relative frequency of the SST anomalies for the 40-year period is shown by dots in Fig. 5, together with the 95% confidence interval estimated from 10,000 bootstrap resampling data. The same histograms were also constructed for the 5 years with thick STMW (1982, 1996, 1997, 2006, and 2015) and the 7 years with thin STMW (1987, 1999, 2001, 2009, 2010, 2020, and 2021), based on fig. S2.

The RSMC Tokyo best track data were used to examine tropical cyclones passing over the southern part of the study region from 1972 to 2021. For tropical depressions or cyclones with the intensity of a tropical storm or higher, we calculated the TCIR at an interval of 6 hours along the track from the difference in the central pressure of a cyclone between 6 hours before and after each time. Here, positive rates denote the development of cyclones with the decrease in pressure with time. The cyclones have been observed frequently from May to November and more intensively in August and September. We first calculated the climatology of the intensification rate in each 2° grid box for May to July, August to September, and October to November. We then computed TCIR anomalies by subtracting the climatology from each value to reduce seasonal and year-to-year biases in the positions of cyclones. In the same manner as SST, we calculated the histograms of the TCIR anomalies for the 50-year period and for the 6 years with thick STMW and the 10 years with thin STMW described above (Fig. 6).

To supplement the results, we also analyzed the Joint Typhoon Warning Center (JTWC) best track data from 1972 to 2021. Instead of central pressure, the maximum sustained wind speed data, which are available over the whole analysis period of the JTWC data, were used to calculate the TCIR. The results are quite consistent with those from the RSMC data (fig. S4).

### Regional atmospheric model experiments

The regional atmospheric model used to simulate Typhoons Lan (2017), Hagibis (2019), and Chan-hom (2020) is the JMA-NHM (*47*, *48*). The JMA-NHM is designed to serve both weather forecasting and atmospheric research needs and has been used in a variety of regional climate studies. The domain covers the study region and the track of typhoons in the Lambert projection, with 5-km horizontal grid spacing (Fig. 7A). The model has 41 vertical levels with realistic topography. We used the Mellor-Yamada-Nakanishi-Niino level 3 planetary boundary layer scheme (*81*), the Beljaars-Holtslag flux and bulk coefficient scheme (*82*), and the Kain-Fritsch convective parameterization scheme (*83*, *84*) for convective processes.

The meteorological initial and boundary conditions were sourced from the National Centers for Environmental Prediction Global Tropospheric final analyses at a grid resolution of 0.25° and an interval of 6 hours. Merged satellite and in situ data Global Daily Sea Surface Temperature, which were provided by the JMA (*85*), were used as the lower boundary condition for the CTRL run. To assess the typhoon response to SSTs in the southern part of the study region (20°N to 30°N, 130°E to 138°E; green rectangle in Fig. 7A), i.e., the area where the STMW has decreased drastically in the current Kuroshio large-meander period (fig. S2), the simulations were initiated before the typhoons entered the target area (see white circles in Fig. 7A). The SST given at the initial time was unchanged throughout the simulations.

To examine the impact of STMW-induced SST changes on the central pressure of typhoons, other experiments were performed under the same meteorological initial and lateral boundary conditions as used for the CTRL run, but with SST anomalies yielded by multiplying STMW thickness anomalies of +50, +100, +150, +200, and +300 dbar by the regression coefficient at 10 dbar in October (−0.56 × 10^−2^°C/dbar; Fig. 4A) being added to the CTRL SST field in the target area. In addition, experiments were conducted with reference to the year 2015, when the STMW thickness was at its peak (fig. S2). The difference in the STMW thickness between October 2015 and October 2017 in the western part of the target area (20°N to 30°N, 130°E to 134°E), where Lan passed through (gray curve in Fig. 7A), was 37 dbar, based on Argo float observations. In the same manner, the difference in the northeastern corner (25°N to 30°N, 135°E to 138°E) and the northern part (25°N to 30°N, 130°E to 138°E) of the target area for Hagibis and Chan-hom were estimated to be 127 and 102 dbar, respectively.

## Supplementary Material

20230913-1

## Notes

**Correction (25 September 2023):** As a result of a production error, the original Fig. 4 included unnecessary green and black lines in panels A and C. The figure has been corrected. The HTML and PDF have been updated.
