# Supplementary material for: Subtropical Mode Water south of Japan impacts typhoon intensity
Source: Sci Adv. 2023 Sep 13;9(37):eadi2793. doi: 10.1126/sciadv.adi2793 (PMC11804680; doi:10.1126/sciadv.adi2793)
Supplement: Supplementary file 2 — Figs. S1 to S4 [file sciadv.adi2793_sm.pdf]

Science Advances
AAAS

Supplementary Materials for

Subtropical Mode Water south of Japan impacts typhoon intensity

Eitarou Oka *et al.*

Corresponding author: Eitarou Oka, eoka@aori.u-tokyo.ac.jp

Sci. Adv. **9**, eadi2793 (2023)
DOI: 10.1126/sciadv.adi2793

This PDF file includes:

Figs. S1 to S4

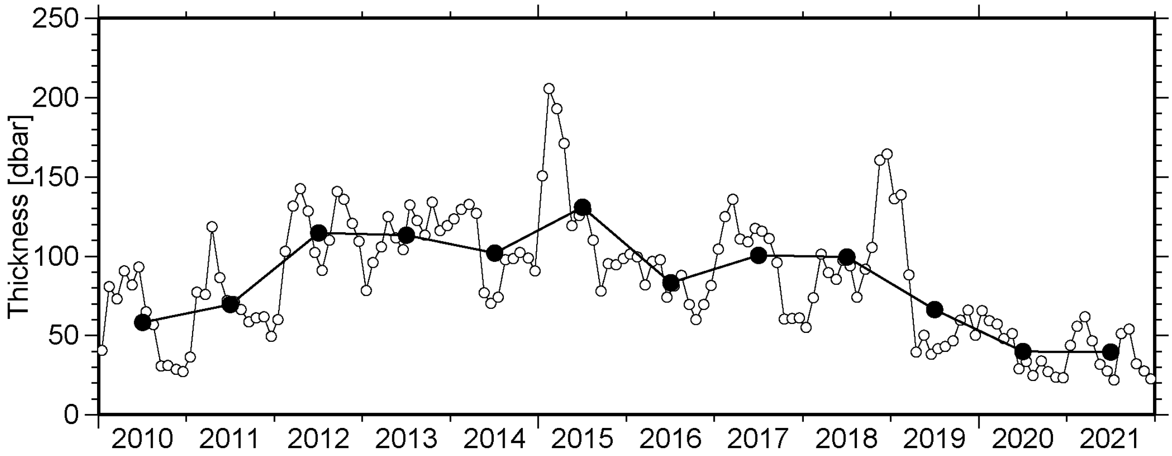

Fig. S1. Argo-based decadal STMW variability in the study region. Monthly STMW thickness averaged in the study region (20º−35ºN, 130º−138ºE; circles) and its yearly average (dots) based on Argo data.

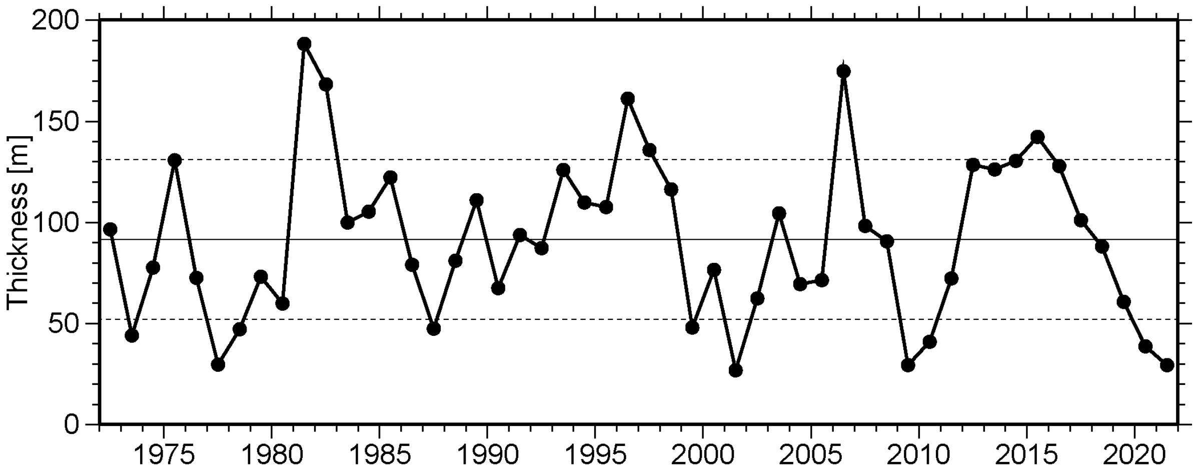

Fig. S2. Decadal STMW variability at 137ºE. STMW thickness in the 137ºE section in summer, averaged at 20º–30ºN. Solid and dashed lines indicate the average and one standard deviation, respectively.

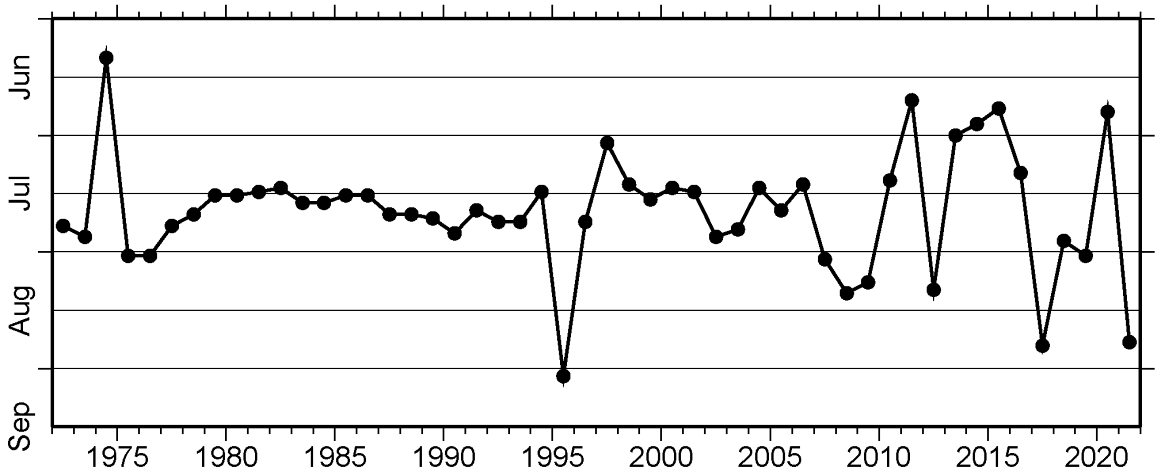

Fig. S3. Observation dates of the 137ºE section in summer. Dates of observation at 25ºN of the 137ºE section in summer. The observations at 20º–30ºN were conducted within ±2 days from this date in most years.

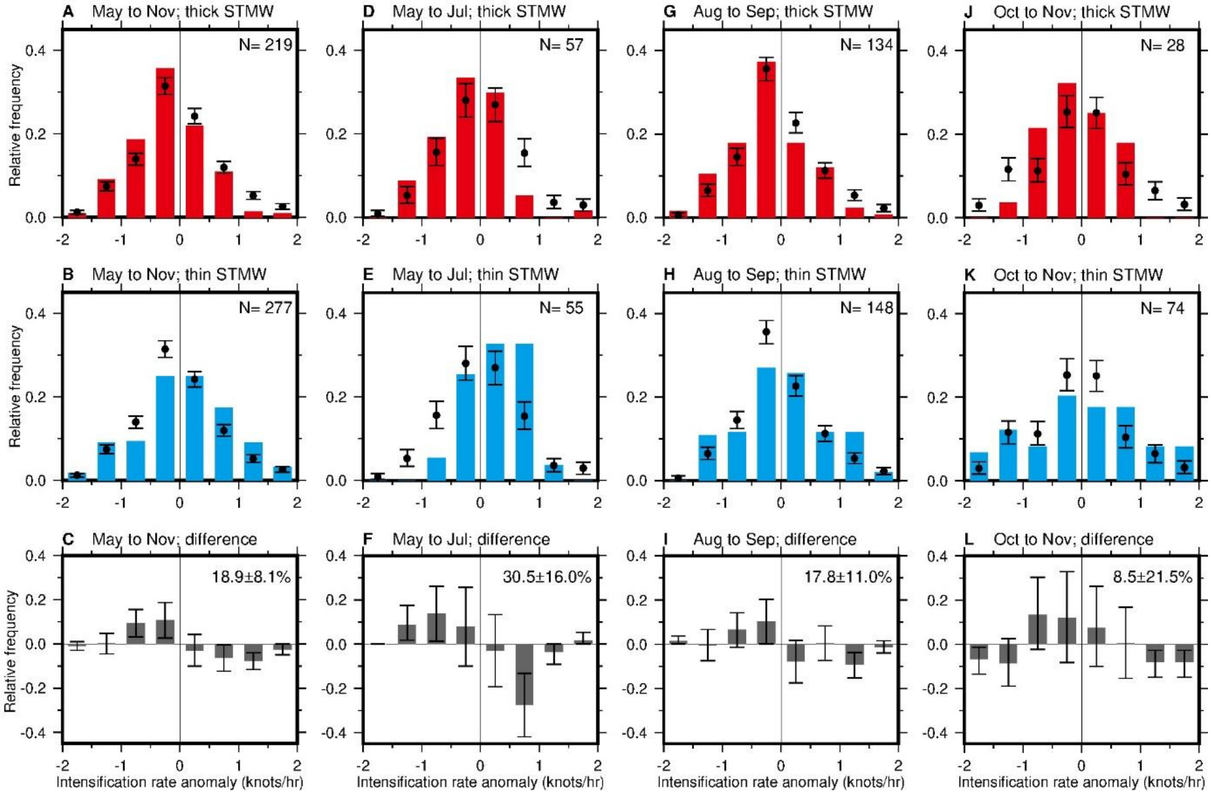

Fig. S4. Observed dependence of the TCIR on the STMW thickness. As for Fig. 5 but for the TCIR anomalies based on the maximum sustained wind speed from the JTWC best track data for the period from 1972 to 2021. The number of data is denoted at the top right corner of each panel in (**A**), (**B**), (**D**), (**E**), (**G**), (**H**), (**J**), and (**K**).
